# The impact of organizational support on turnover intentions among novice teachers in primary and secondary schools of less developed areas: the sequential mediating role of induction adaptation and job burnout

**DOI:** 10.3389/fpsyg.2026.1736702

**Published:** 2026-03-18

**Authors:** Xiuwei Yang, Lili Lu, Duoxiu Ma

**Affiliations:** 1 Faculty of Education, Guangxi Normal University, Guilin, Guangxi, China; 2 Liping County Secondary Vocational School, Liping, Guizhou, China; 3 Baoji University of Arts and Sciences, Baoji, Shaanxi, China

**Keywords:** induction adaptation, job burnout, organizational support, teacher, turnover intention

## Abstract

Imbalanced educational development is a matter of widespread concern across the globe, and novice teacher turnover is a common phenomenon in primary and secondary schools within less-developed regions. Identifying the factors contributing to these teachers' turnover intentions is crucial to addressing the challenge of educational inequality. To investigate the impact pathways and mechanisms of organizational support on turnover intentions among novice teachers in primary and secondary schools of less developed areas, data were collected from 520 primary and secondary school teachers within five years of joining the profession in 3 counties of Guizhou Province, China. The results indicate that 44% of novice teachers in primary and secondary schools have turnover intention at a medium level or above, and the potential problem of “unable to retain” is still more prominent. Organizational support significantly and positively predicted novice teachers' turnover intentions. Both induction adaptation and job burnout demonstrated independent and sequential mediating effects between organizational support and turnover intention. Notably, while induction adaptation directly and negatively predicted turnover intention (β = −0.346, *p* < 0.001), it exhibited a positive mediating effect (*b* = 0.144) after controlling for the influence of job burnout. This suggests that induction adaptation may play a “double-edged sword” role in the relationship between organizational support and turnover intention. These findings help reconcile the debate regarding the cross-cultural applicability of the “dual-effect” of induction adaptation, providing new evidence and perspectives for both research on and interventions addressing novice teacher turnover.

## 1 Introduction

Improving educational quality and building a strong educational nation not only concerns individual development, public welfare, and social justice, but also relates to enhancing a country's comprehensive strength, consequently, it has become a strategic choice for nations in their socioeconomic and technological competition (Li et al., 2025). Building a high-quality, professional teaching force is a key prerequisite for enhancing educational quality, and the stable, orderly development of novice teachers serves as the driving force behind strengthening this professional workforce. However, teacher attrition rates are typically highest among those in their first five years of teaching (Fantilli and McDougall, 2009; Macdonald, 1999). A survey on rural teachers in China found that those with three to 5 years of teaching experience exhibit the strongest intention to leave the profession (Wang et al., 2022). A longitudinal study in the United States revealed that 45% of teachers depart the teaching profession within 5 years of entering it (Ingersoll et al., 2018). Similar high attrition rates among early-career teachers have also been observed in countries such as the United Kingdom (Department for Education, 2019), Chile (Ávalos and Valenzuela, 2016), and Belgium (Dupriez et al., 2016). Teacher attrition has thus become a global issue (OECD, 2006).

Existing studies on novice teacher attrition have primarily focused on groups such as beginning kindergarten teachers, elementary school novices, and young rural teachers, examining antecedent variables of turnover intention including individual characteristics, contextual factors, social support, and job burnout (Redding and Nguyen, 2020; Tiplic et al., 2015; Goddard and Goddard, 2006). For instance, studies indicate that new teachers mentored by peers within the same subject area and participating in collective induction activities—such as collaborative lesson planning with other educators—are less likely to change schools during their second year of employment or abandon the teaching profession (Smith and Ingersoll, 2004). Furthermore, organizational trust exerts a negative influence on kindergarten teachers' turnover intention, mediated through the chain mediating effects of teaching efficacy and job satisfaction (Zhao et al., 2022). However, evidence-based research on these issues remains far from sufficient. On one hand, studies on new teacher turnover in primary and secondary schools in underdeveloped regions are notably scarce. On the other hand, the relationships between new teachers' induction adaptation and job burnout or turnover intention, as well as incorporating induction adaptation into frameworks examining organizational support, job burnout, and turnover intention, has received less scholarly attention. In fact, novice teachers not only face survival challenges in induction adaptation but also exhibit pronounced job burnout (Gavish and Friedman, 2010).

According to the Conservation of Resources (COR) theory, which posits that individuals strive to acquire, maintain, cultivate, and protect their personal resources (Hobfoll, 1989), novice teachers who face continuous challenges at work and struggle to adapt, without receiving effective organizational support to prevent resource depletion, may become trapped in a “loss spiral”. This situation leads to heightened stress, the development of job burnout, and an increased likelihood of turnover intention. Therefore, studying the impact of organizational support on turnover intention among novice teachers in underdeveloped regions, with induction adaptation and job burnout as mediating variables, holds significant theoretical and practical implications.

### 1.1 The impact of organizational support on the turnover intentions among novice teachers in primary and secondary schools of less developed regions

Turnover intention refers to the desire or inclination of organizational members to depart their current institution following an assessment of resignation expectations and costs, prompted by internal and external factors. It constitutes the final and critical stage in the decision-making mechanism for resignation behavior (Mobley, 1977). Novice teacher turnover intention refers to the psychological inclination among early-career teachers—in this study, defined as those within their first 5 years of teaching in primary or secondary schools—to depart their current positions due to dissatisfaction with their working conditions. This encompasses both mobility turnover intention (seeking roles at other schools or educational institutions) and attrition turnover intention (transitioning to different industries). Increased turnover intentions among novice teachers are often accompanied by diminished work performance, which not only hinders students' healthy development but also imposes self-imposed limitations and delays on teachers' professional growth. Since teachers can only advance to higher stages in professional development when they fully commit to teaching without hesitation or consideration of alternative careers.

In the 1980s, American social psychologist Eisenberger developed the Organizational Support Theory by expanding on concepts such as the “norm of reciprocity” and the “personification of the organization (Eisenberger et al., 1986).” Organizational support refers to an organization's emphasis on members' contributions and welfare, alongside providing sustained assistance for their career development needs. Members' overall perception of organizational support constitutes a crucial factor in their willingness to remain within the organization and contribute to it. Research indicates that schools with departing novice teachers demonstrate significantly lower levels of organizational support—including principal leadership, mentor influence, and facilities and resources—compared to those retaining early-career teachers (Redding and Henry, 2019). In other words, inadequate organizational support serves as a critical factor contributing to novice teacher attrition. Consequently, we hypothesize that organizational support can significantly reduce turnover intentions among novice teachers in primary and secondary schools in less-developed regions.

### 1.2 The mediating role of induction adaptation

Career adaptation refers to an individual's self-regulatory strengths or capabilities employed to solve unfamiliar, complex, and uncertain problems arising from developmental career tasks, occupational transitions, and work-related setbacks (Savickas and Porfeli, 2012). Induction adaptation of novice teachers denotes the self-regulatory and restorative capacity of early-career teachers when confronting role transitions, professional responsibilities, workplace frustrations, and unforeseen events that alter career plans. During this adaptive process, novice teachers inevitably experience “reality shock” when discrepancies emerge between their expectations and the actualities of professional anticipation, work environment, student circumstances, interpersonal relationships, and theoretical vs. practical skills. Based on a synthesis of existing literature, Veenman (1984) summarized five potential outcomes triggered by reality shock: perceptions of problems, Changes of behavior, Changes of attitudes, Changes of personality, and Leaving the teaching position. Thus, in the short term, these outcomes may lead to physical and psychological discomfort, as well as alterations in educational beliefs and practices among novice teachers. In the long term, they can negatively impact professional identity and increase turnover intention. Lee et al.'s employee turnover model (Lee et al., 1999) indicates that the reality shock not only triggers existing resignation experiences and action plans among new teachers but also creates conflicts in their values and objectives with the institution. This leads them to evaluate and compare their current role with alternative employment opportunities, thereby fostering intention to leave. Research indicates that teachers' career adaptability, akin to teaching self-efficacy, can mitigate emotional exhaustion and resignation intentions among educators (Wang et al., 2015). Concurrently, studies examining factors influencing onboarding adaptation suggest that providing support and guidance to new teachers facilitates their successful adaptation to the role (Haritos, 2004). Therefore, we postulate that induction adaptation may serve as a negative mediator between organizational support and turnover intention. That is, organizational support in less-developed regions may negatively influence novice teachers' turnover intentions through the mediating effect of their induction adaptation.

### 1.3 The mediating role of job burnout

Teacher job burnout refers to a state of exhaustion manifested in physical, psychological, and behavioral dimensions due to doubts about professional value and personal competence. It encompasses three dimensions: emotional exhaustion, depersonalization, and personal accomplishment, and is regarded as a form of work-related stress associated with variables such as job satisfaction, organizational commitment, and staff turnover (Maslach et al., 2001). Regarding the development of job burnout, the Job Demands-Resources model posits that an imbalance between job demands and job resources often leads to feelings of fatigue and burnout, thereby triggering job burnout and turnover (Schaufeli and Taris, 2014). An empirical study focusing on early-career teachers within their first 2 years of service in Australia demonstrated that job burnout significantly and positively predicts turnover intention (Goddard and Goddard, 2006). A meta-analytic study also concluded that job burnout is the most important predictor of teachers' turnover intentions (Li and Yao, 2022). Thus, it can be postulated that Job burnout plays a negative mediating role between organizational support and turnover intention. In other words, organizational support in less-developed regions can reduce novice teachers' turnover intention by curbing their job burnout.

### 1.4 The sequential mediating role of induction adaptation and job burnout

If newly appointed teachers can adapt to the difficulties and challenges arising from shifts in identity, context, and practice, they will develop a positive professional experience and enhance their sense of teaching efficacy, thereby avoiding job burnout. Consequently, induction adaptation may be a significant factor influencing job burnout. According to COR theory (Hobfoll, 2001), the process of interaction between individuals and their environment generates two opposing pathways: resource gain and resource depletion. The resource gain pathway occurs when individuals receive material, conditional, or psychological resources exceeding their investment in response to work demands. This continuous resource accumulation strengthens career adaptation, increases work engagement, reduces burnout, and fosters positive outcomes such as high organizational commitment and low turnover intention. Conversely, the resource depletion pathway occurs when individuals receive fewer returns than their input. Over time, this creates “loss spirals of resources”, weakening work adaptation, inducing work anxiety, triggering job burnout, and subsequently leading to negative outcomes such as low organizational commitment and high turnover intention. Extensive empirical research indicates that teachers' induction adaptation directly and negatively influences work burnout (Chen et al., 2025). Compared to those who leave, retained new teachers typically demonstrate stronger induction adaptability. They effectively manage the gap between reality and expectations, bridge the discrepancy between personal knowledge and skills with job requirements, and overcome challenges in organizational cultural integration and interpersonal communication, ultimately avoiding burnout (Hong, 2010). Based on this, the study proposes that induction adaptation and job burnout may sequentially mediate the effect of organizational support on novice teachers' turnover intentions.

### 1.5 The current study

**Figure 1:**
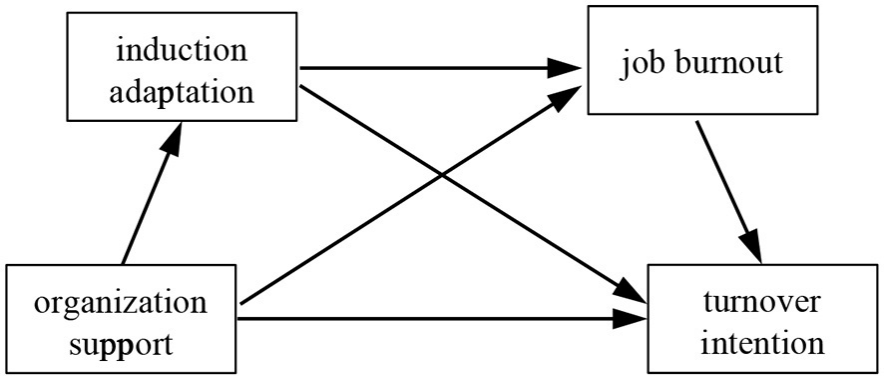
The proposed model of the current study.

Based on the aforementioned literature analysis, there may be close interrelationships among organizational support, induction adaptation, job burnout and turnover intention. Organizational support theory suggests that perceived organizational support enhances employees' felt obligation to help the organization achieve its goals and increases their affective organizational commitment, thereby reducing turnover and other withdrawal behaviors (Eisenberger et al., 2002). When employees lack organizational support and fail to obtain necessary resources, their level of career adaptation will be adversely affected (Wang et al., 2024). Teachers' level of career adaptation, in turn, serves as a predictor of their degree of burnout (Jaoul and Kovess, 2004; Carstensen and Klusmann, 2021), which is the leading reason of turnover among educators (Schaack et al., 2020; Russell et al., 2020). According to the COR theory, individuals tend to seek to acquire and preserve personal resources (Hobfoll, 1989). If such resources are continuously threatened by potential or actual loss, individuals will encounter career adaptation problems and experience emotional exhaustion, ultimately leading to turnover intentions and behaviors (Schaufeli et al., 2018). Accordingly, the current study conducted a questionnaire survey among novice teachers across three counties in Guizhou Province, China, aiming to explore the relationship between organizational support and turnover intentions among novice teachers in primary and secondary schools within less-developed regions, and to indicate the independent and sequential mediating effects of induction adaptation and job burnout in this relationship. On this basis, four research hypotheses and the corresponding theoretical model are proposed as follows (Figure 1).

**Hypothesis 1**. Organizational support significantly predicts turnover intention among novice teachers in primary and secondary schools within underdeveloped regions.

**Hypothesis 2**. Induction adaptation plays a negative mediating role between organizational support and turnover intention.

**Hypothesis 3**. Job burnout plays a negative mediating role between organizational support and turnover intention.

**Hypothesis 4**. Induction adaptation and sequentially mediate the relationship between organization support and turnover intention.

## 2 Methods

### 2.1 Participants and procedures

Based on socioeconomic and geographical factors, this study selected novice teachers with less than 5 years of teaching experience from D County, X County, and K City in Guizhou Province, representing less-developed regions. A random sampling method was implemented through an online questionnaire survey. A total of 538 responses were collected, and after excluding questionnaires with excessively short completion times or over 90% identical answers, 520 valid responses were retained, resulting in a valid response rate of 96.7%. The valid sample comprised teachers from various school types and locations: 57 (11.0%) from high schools, 49 (9.4%) from urban middle schools, 137 (26.3%) from township middle schools, 106 (20.4%) from urban primary schools, 140 (26.9%) from township primary schools, and 31 (6.0%) from village schools. The sample was predominantly characterized by female (78.7%), unmarried (62.3%), under 30 years old (83.8%), holding bachelor's degrees (96.7%), and tenured teachers (87.1%).

Regarding the data collection process, samples from D County were obtained through on-site data collection during a training session for novice teachers with less than 5 years of experience. After obtaining consent from the session organizers, the researcher explained the survey instructions and administered the questionnaire collectively to the participating teachers. In contrast, samples from X County and K City were primarily collected through online questionnaires distributed to school teachers with the consent and facilitation of education administration department leaders and school principals. During the data collection process, the research followed the ethical standards of studies, such as anonymity, confidentiality, and voluntary participation.

### 2.2 Measures

#### 2.2.1 Organizational support

The Perceived Organizational Support Scale, adapted from the instrument developed by Kottke and Sharafinski (1988), was used to assess organizational support. It comprises nine items across three dimensions: work support, value recognition, and concern for interests. Participants were asked to report on a 5-point scale ranging from 1 (“strongly disagree”) to 5 (“strongly agree”), with higher scores indicating higher levels of organizational support. In this study, the Cronbach's α coefficient of the overall scale was 0.929, and the coefficients for the work support, value recognition, and concern for interests subscales were 0.798, 0.812, and 0.926, respectively. Confirmatory factor analysis demonstrated acceptable model fit: χ^2^/*df* = 2.443, RMSEA = 0.077, CFI = 0.976, IFI = 0.976, TLI = 0.964, demonstrating that the scale has satisfactory reliability and validity.

#### 2.2.2 Induction adaptation

The Induction Adaptation Questionnaire, adapted from the version modified by Lu and Yin (2016), was used to assess novice teachers' adaptation. It contains 12 items (e.g., “I can effectively maintain order and exercise appropriate control when organizing group activities for students”) measuring three dimensions: teaching skills, interpersonal relationships, and psychological readiness. Responses were recorded on a 5-point scale ranging from 1 (“strongly disagree”) to 5 (“strongly agree”), with higher total scores indicating better induction adaptation. In this study, the Cronbach's α coefficient of the overall scale was 0.920, and the coefficients for the teaching skills, interpersonal relationships, and psychological readiness subscales were 0.849, 0.794, and 0.878, respectively. Confirmatory factor analysis indicated acceptable model fit: χ^2^/*df* = 3.322, RMSEA = 0.097, CFI = 0.936, IFI = 0.936, TLI = 0.917, the scale demonstrated good reliability and validity.

#### 2.2.3 Job burnout

Job burnout was assessed using the Maslach Burnout Inventory (MBI) (Maslach and Jackson, 1981) and its revised version for Chinese teachers by Xin-Chun et al. (2016). The scale comprises 22 items across three dimensions: emotional exhaustion, depersonalization, and reduced personal accomplishment. Participants were required to self-report on a 5-point scale ranging from 1 (“never”) to 5 (“always”). After reverse-scoring the items in the reduced personal accomplishment dimension, higher total scores indicate higher levels of job burnout. In this study, the Cronbach's α coefficient of the overall scale was 0.892, and the coefficients for the emotional exhaustion, depersonalization, and reduced personal accomplishment subscales were 0.947, 0.909, and 0.733, respectively. CFA confirmed good model fit: χ^2^/*df* = 1.832, RMSEA = 0.057, CFI = 0.949, IFI = 0.949, TLI = 0.943, demonstrating that the MBI possesses good reliability and validity.

#### 2.2.4 Turnover intention

The Turnover intention Scale, adapted from Mobley et al. (1978), was used to assess participants' inclination to leave their current positions. The scale consists of 4 items (e.g., “I often think about leaving my current school”) rated on a 5-point scale ranging from 1 (“strongly disagree”) to 5 (“strongly agree”). Higher total scores indicate stronger turnover intention. In this study, the Cronbach's α coefficient was 0.892. Following model revision, the CFA demonstrated excellent model fit: χ^2^/*df* = 2.409, RMSEA = 0.001, CFI = 0.999, IFI = 0.999, TLI = 0.990. The scale demonstrated good reliability and validity.

#### 2.2.5 Control variables

Consistent with previous research findings, this study controlled for the following demographic and occupational variables: gender (1 = male, 2 = female), marital status (1 = unmarried, 2 = married), age (1 = under 30 years, 2 = 30 years or above), and professional title (1 = not yet rated, 2 = primary level or above). Two sets of dummy variables were created to account for positional and institutional differences:

Position type: with “regular teacher” as the reference group, categories included homeroom teacher and department head (including grade-level or subject group leaders).

School type: with “high school” as the reference group, categories included urban middle school, township middle school, urban primary school, township primary school, and village school (including teaching sites).

The specific items of the above four scales—Organizational Support, Induction Adaptation, Job Burnout, Turnover Intention—are shown in Table 1.

**Table 1:** Summary of scales and items.

**Variable**	**Dimension**	**Item content**
Organizational Support	Work support	I receive timely help when I encounter problems at work.
		School leaders notice teachers with excellent job performance.
		The school provides opportunities for promotion.
	Value recognition	The school provides opportunities for promotion.
		I am proud to be a member of this school.
		School leaders take pride in my achievements.
	Concern for interests	The school takes teachers' interests into account when making decisions.
		School leaders care about teachers' wellbeing and living conditions.
		The school recognizes teachers' contributions in certain ways.
Induction adaptation	Teaching skills	I can correctly understand instructional objectives and select appropriate teaching content based on instructional needs.
		I will adopt different teaching methods for different educational contents and activity links.
		I can maintain order well and exercise appropriate control over the activities when organizing students' collective activities.
		I can use the school's teaching equipment proficiently.
	Interpersonal relationships	I can get practical help from colleagues when I encounter teaching problems.
		I am good at handling relationships with students' parents.
		I can respect and support the arrangements of educational administrators.
		I am able to establish a harmonious teacher–student relationship with my students.
	Psychological readiness	I have clear goals and plans in my work at the school.
		I clearly know what efforts I still need to make to achieve my development goals.
		The mentorship or support measures arranged by the school for me as a new teacher help me adapt to the profession more quickly.
		I can quickly adjust my mindset and devote myself to my current work.
Job burnout	Emotional exhaustion	I feel burned out from my work.
		I feel frustrated by my job as a teacher
		I feel used up at the end of the workday
		I feel emotionally drained from my teaching work
		I feel tense and stressed throughout the workday
		I feel fatigued when I get up in the morning
		I feel emotionally drained from my work.
		I feel I am working too hard on my job
	Personal accomplishment	I can easily create a relaxed atmosphere with my students
		I have accomplished many worthwhile things in this job
		I can help my students gain self-confidence.
		I deal very effectively with the problems of my students
		I can provide my students with useful guidance
		I feel I am positively influencing other peoples lives through my work.
		My students are willing to talk with me when they have problems
		I can easily understand how my students feel
	Depersonalization	I snap at students over minor things
		I am harsh with my students
		I have the urge to yell at my students
		I feel my students are dissatisfied with how I handle problems
		I feel I treat some students as if they were impersonal objects
		I seldom praise my students
Turnover intention		I would leave my current position if a more ideal job became available.
		I strongly desire to find a job that is more ideal than my current one.
		I will leave my current school within the next year or two.
		I often think about leaving my current school.

### 2.3 Data analysis

Data analysis was conducted using statistical software SPSS 26.0 and AMOS 26.0. First, SPSS 26.0 was employed to perform analyses for internal consistency (Cronbach's α), common method bias, descriptive statistics, and variables correlations. Meanwhile, AMOS was used to examine the validity of the four main scales. Subsequently, a mediation model was established. The SPSS PROCESS 4.1 was utilized to examine the relationship between organizational support and novice teachers' turnover intentions, as well as the mediating roles of induction adaptation and job burnout in this relationship. The significance of the mediating effects was tested using the bootstrapping method.

## 3 Results

### 3.1 Common method bias test

This study employed Harman's one-factor test to assess the potential presence of common method bias. The results revealed that factor analysis identified seven eigenvalues greater than 1, with the first principal component explaining 32.49% of the total variance—below the critical threshold of 40%. This indicates no significant common method bias in the current study.

### 3.2 Descriptive statistics and correlations

According to the descriptive statistics presented in Table 2, the mean scores for organizational support and induction adaptation were 3.500 and 3.898 respectively, both falling within the moderate range (based on the 5-point scale with 3 as the midpoint). Job burnout showed a mean score of 2.599, while turnover intention averaged 2.632, below the moderate level. However, further analysis revealed that 44% of novice teachers demonstrated moderate to high levels of turnover intention, with 35% explicitly stating they “would leave if a more ideal job opportunity became available”.

**Table 2:** Results of descriptive statistics and correlations (*N* = 520).

**Variable**	**M**	**SD**	**1**	**2**	**3**	**4**
1.Organizational support	3.500	0.753	1			
2.Induction adaptation	3.898	0.569	0.595^**^	1		
3.Job burnout	2.599	0.538	−0.543^**^	−0.584^**^	1	
4.Turnover intention	2.632	1.123	−0.581^**^	−0.370^**^	0.611^**^	1

^**^p < 0.01.

Correlation analysis indicated that among the control variables, marital status, age, and school type showed significant correlations with core variables, thus warranting statistical control in subsequent analyses. Regarding the four core variables: organizational support showed a significant positive correlation with induction adaptation (*r* = 0.569, *p* < 0.01), while demonstrating significant negative correlations with both job burnout (*r* = −0.543, *p* < 0.01) and turnover intention (*r* = −0.581, *p* < 0.01); induction adaptation correlated negatively with both job burnout (*r* = −0.584, *p* < 0.01) and turnover intention (*r* = −370, *p* < 0.01); and job burnout was positively correlated with turnover intention (*r* = 0.611, *p* < 0.01). These significant correlations among key variables provide preliminary evidence for further hypothesis testing.

### 3.3 Hypothesis test

First, multiple linear regression analyses were conducted for pairwise combinations of variables to test the direct effects. As shown in Table 3, after incorporating control variables, Model 2 demonstrated that organizational support had a significant negative effect on turnover intentions among novice teachers in underdeveloped regions (β = −0.563, *p* < 0.001), supporting Hypothesis H1. Models 3 and 4 revealed that organizational support significantly positively influenced induction adaptation (β = 0.591, *p* < 0.001), while induction adaptation significantly negatively influenced turnover intention (β = −0.346, *p* < 0.001), suggesting that induction adaptation may mediate the relationship between organizational support and turnover intention. Models 5 and 6 indicated that organizational support significantly negatively influenced job burnout (β = −0.524, *p* < 0.001), and job burnout significantly positively influenced turnover intention (β = 0.598, *p* < 0.001), suggesting that job burnout may also mediate the relationship between organizational support and turnover intention. Model 7 showed that induction adaptation significantly negatively influenced job burnout (β = −0.561, *p* < 0.001). In summary, these results indicate that induction adaptation and job burnout may sequentially mediate the relationship between organizational support and turnover intention.

**Table 3:** Results of multiple linear regression analyses (*N* = 520).

**Variable**	**Model 1 TI**	**Model 2 TI**	**Model 3 IA**	**Model 4 TI**	**Model 5 JB**	**Model 6 TI**	**Model 7 JB**
Marital status	−0.072	−0.067	0.061	−0.049	−0.081^*^	−0.021	−0.048
Age	−0.113^*^	−0.099^**^	0.031	−0.097^*^	−0.001	−0.105^**^	0.011
**School type (reference: high school)**							
Urban middle school	0.003	0.028	−0.03	0.002	0.123^*^	−0.057	0.098^*^
Township middle school	0.071	0.028	−0.031	0.045	0.172^**^	−0.055	0.169^**^
Urban primary school	−0.106	−0.05	0.025	−0.077	0.005	−0.077	0
Township primary school	0.074	0.049	0.078	0.092	0.073	0.016	0.125^*^
Village school	−0.101	−0.073	0.048	−0.074	−0.035	−0.064	−0.018
OS		−0.563^***^	0.591^***^		−0.524^***^		
IA				−0.346^***^			−0.561^***^
JB						0.598^***^	
*R* ^2^	0.060	0.367	0.374	0.175	0.332	0.394	0.370
Δ*R*^2^	0.060	0.308	0.339	0.115	0.267	0.334	0.304
*F*	4.660^***^	37.106^***^	38.158^***^	13.566^***^	31.799^***^	41.595^***^	37.532^***^
DW	1.939	1.919	1.902	1.917	2.003	1.920	2.057

^*^p < 0.05.

^**^p < 0.010.

^***^p < 0.001.

OS, organizational support; TI, turnover intention; IA, induction adaptation; JB, job burnout.

**Figure 2:**
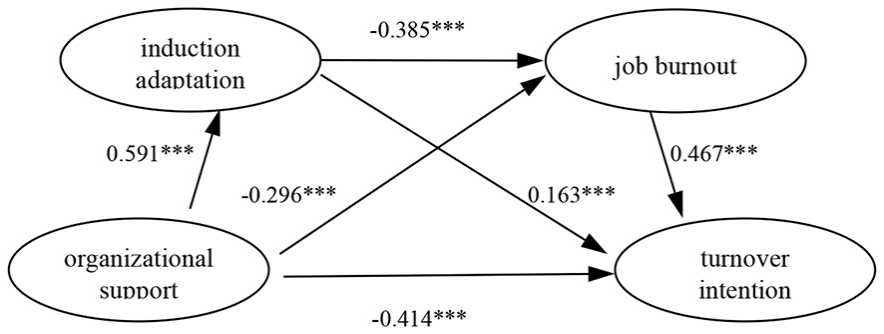
Result of sequential model test. ***p < 0.001.

Secondly, the predefined Model 6 in the SPSS Process 4.1 plug-in was employed to examine the predictive effects among variables, with the Bootstrap method (5,000 bootstrap resamples) used to test the significance of mediating effects. The standardized path coefficients derived from Process analysis are presented in Figure 2. The results indicate significant mediating roles of both induction adaptation and job burnout. As shown in Table 4, Bootstrapping analyses revealed that the direct effect of organizational support on turnover intentions among novice teachers in underdeveloped regions was significantly negative (*b* = −0.618, *p* < 0.001, 95%CI [−0.740, −0.496]), confirming that the model is a partial mediation model.

**Table 4:** Bootstrapping results of indirect effects of the sequential mediation model (*N* = 520).

**Pathways**	**Effects**	**SE**	**95%CI**		**Percent**
			**LL**	**UL**	
Total effect	−0.839	0.053	−0.944	−0.735	
Direct effect	−0.618	0.062	−0.740	−0.496	73.7%
Total indirect effect	−0.221	0.045	−0.314	−0.139	26.3%
OS → IA → TI	0.144	0.040	0.069	0.224	
OS → JB → TI	−0.206	0.035	−0.281	−0.140	
OS → IA → JB → TI	−0.159	0.026	−0.212	−0.113	

OS, organizational support; IA, induction adaptation; JB, job burnout; TI, turnover intention; SE, standard error; CI, confidence interval; LL, lower limit; UL, upper limit.

Among the three potential indirect pathways through which organizational support influences turnover intention, indirect Path 1(OS → IA → TI): the indirect effect was 0.144, with a 95% CI [0.069, 0.224] that does not contain zero. This finding indicates that induction adaptation exerts a significant positive indirect effect on the relationship between organizational support and turnover intention. Contrary to the hypothesized direction, this suggests that while organizational support enhances induction adaptation, such improvement may concurrently be associated with increased turnover intention rather than consistently reducing it. Therefore, Hypothesis H2 was not fully supported.

Indirect Path 2(OS → JB → TI): the indirect effect was −0.206, with a 95% CI [−0.281, −0.140] excluding zero. This finding confirms that job burnout serves as a significant negative mediator in this relationship, supporting Hypothesis H3.

Indirect Path 3(OS → IA → JB → TI): the indirect effect was −0.159, with a 95% CI [−0.212, −0.113] excluding zero. This finding indicates the chained mediating role of induction adaptation and job burnout in the relationship between organizational support and turnover intention, supporting Hypothesis H4.

Based on the foregoing analysis, a suppressing effect exists in the relationship between organizational support and turnover intention. After introducing induction adaptation and job burnout as mediating variables, the direct effect of induction adaptation on turnover intention shifted from negative (β = −0.346, *p* < 0.001) in the initial regression model to positive (β = 0.163, *p* < 0.001). Meanwhile, the indirect path of induction adaptation through job burnout (β = −0.385 × 0.467 = −0.180) exerted a negative influence on turnover intention. These two opposing pathways constitute a suppressing effect, accounting for 110.3% (|−0.385 × 0.467/0.163|). This indicates that the positive impact of induction adaptation is partially masked by its negative effect achieved through reducing job burnout, resulting in an overall negative indirect effect (−0.221).

## 4 Discussion

This study investigated 520 novice teachers from primary and secondary schools across three counties in Guizhou Province, a less-developed region of China, aiming to explore the direct impact of organizational support on turnover intentions and its underlying mediating mechanisms. The findings reveal that organizational support not only directly predicts novice teachers' turnover intentions but also indirectly influences them through both the independent and sequential mediating roles of induction adaptation and job burnout. This research provides new perspectives and insights for both theoretical research and practical interventions addressing the persistent challenge of teacher turnover.

### 4.1 Direct effect of organizational support on turnover intentions

Descriptive statistics reveal that the level of organizational support perceived by novice teachers in primary and secondary schools within underdeveloped regions remains moderate (*M* = 3.500). Although their turnover intentions fall below the moderate level (*M* = 2.632), significant group differentiation exists: 44% of novice teachers exhibited moderate to high levels of turnover intention. Regarding the statement, “I would leave if a more ideal job opportunity arose,” 31.7% of novice teachers expressed uncertainty, while 35% provided a definitive affirmative response. This indicates that the issue of turnover propensity remains concerning, with the potential problem of “unable to retain staff” is still prominent.

Furthermore, the results demonstrate that organizational support significantly and negatively predicts turnover intentions among novice teachers in these regions. This finding aligns with Organizational Support Theory, which posits the “norm of reciprocity” as a core principle (Rhoades and Eisenberger, 2002). When employees perceive that the organization values their contributions, cares for their wellbeing, and supports their professional development, they experience positive affective responses and a sense of obligation to reciprocate. This, in turn, motivates behaviors beneficial to the organization, such as increased engagement, improved performance, and a greater willingness to remain with the organization. Supporting this, a study based on data from North Carolina, USA, indicated that higher rates of novice teacher mobility—whether within the education system or through leaving the profession altogether—are often observed in schools characterized by inadequate support (Redding and Henry, 2019). Similarly, research focusing on rural kindergarten teachers in China highlighted that organizational support—manifested through recognition, acceptance, and respect—partially fulfills teachers' socio-emotional and psychological needs. Consequently, it serves as a significant negative predictor of turnover intentions among rural kindergarten teachers (Wang et al., 2023).

### 4.2 Mediating role of induction adaptation

The study found that induction adaptation significantly and negatively predicted turnover intention in the initial model (β = −0.346, *p* < 0.001). However, after introducing job burnout as a mediating variable, this path coefficient significantly reversed to positive (β = 0.163, *p* < 0.001), indicating that job burnout exerted a suppressing effect. This suggests that while induction adaptation can reduce job burnout, in certain contexts, higher levels of induction adaptation may also be associated with increased turnover motivation, warranting further investigation into its underlying mechanisms.

Induction adaptation is inherently a form of career adaptation, and its impact on turnover intention remains debated in academia. Studies supporting a positive relationship argue that career adaptation is a “double-edged sword”: it can enhance affective commitment but may also reduce an individual's dependency on the organization and increase alternative job opportunities, thereby raising turnover intention (Ito and Brotheridge, 2005; Klehe et al., 2011). Conversely, research supporting a negative relationship indicates that career adaptability is negatively correlated with turnover intention (Haibo et al., 2017), with kindergarten teachers' career adaptability significantly and negatively influencing their turnover intentions (Zhang and Nan, 2020). The divergence in conclusions may be attributed to cross-cultural differences. Studies reporting positive effects often originate from contexts outside China, where samples are characterized by higher occupational instability and uncertainty. This study supports the “double-edged sword” perspective but reveals that the overall effect of induction adaptation on turnover intention remains predominantly negative. This finding diverges from the simplistic positive-negative dichotomy, demonstrating empirically that its influence is not a clear-cut opposition but rather a complex, dynamic process involving bidirectional interactions. Specifically, the predictive effect of induction adaptation on turnover intention is suppressed by negative emotional experiences such as job burnout. Within an overall negative relationship, a localized positive effect manifests as a “suppression” phenomenon.

A plausible explanation is that individuals with strong induction adaptation often possess higher competencies, potentially making them better equipped and more confident in seeking ideal alternative employment (Orie and Semeijn, 2022). When faced with negative emotional experiences or occupational uncertainty, they may be more inclined to consider leaving. For instance, heavy workloads, complex student situations, and feelings of isolation and lack of support are frequently identified as critical factors contributing to the turnover of novice teachers (Zhang et al., 2023). On the other hand, novice teachers are still exploring various possibilities in adult life (Levinson et al., 1979), with relatively more job options and lower continuous commitment, amid existing uncertainties. This inadvertently creates an environment similar to the contexts where positive effects were observed. In China, many new teachers are not content to remain in their positions. For instance, influenced by traditional notions, civil service positions are highly favored by young teachers, and taking the civil service examination has become one of the key push factors for their turnover intentions and behaviors (Zhou and Xie, 2024; Wang and Feng, 2020; Zhang, 2019). Some studies refer to the phenomenon where the original predictive direction reverses after introducing a new variable as “cross-over suppression” (Paulhus et al., 2004). This study provides a new perspective for uncovering the “black box” of how the impact of induction adaptation on turnover intention dynamically shifts from negative to positive influence.

### 4.3 Mediating role of job burnout

The study reveals that job burnout plays a significant negative mediating role in the relationship between organizational support and novice teachers' turnover intentions. This mediating mechanism can be explained by the Job Demands-Resources (JD-R) model (Schaufeli and Taris, 2014). Novice teachers in the “survival concern” stage are particularly vulnerable: when intense and prolonged job demands—such as classroom management, instructional design, student counseling, and interpersonal communication—consistently impair their wellbeing, job burnout is likely to occur. Conversely, when organizational support provides sufficient job resources that reduce the physical and psychological costs of meeting these demands, the resulting motivational process can alleviate teacher burnout. Job burnout reflects negative emotional experiences, physical and mental depletion, and impeded professional growth, often leading individuals to rationally consider leaving the profession. Research has confirmed that job resources can suppress job burnout, and job burnout significantly mediates the relationship between job resources and turnover intention (Kaiser et al., 2020). Interviews with former teachers who have left the profession indicate that negative work experiences and perceived career development bottlenecks are primary drivers of teacher attrition. Consequently, mitigating job burnout to reduce turnover intention has become a critical issue in building a stable and high-quality teaching force in primary and secondary schools within less-developed regions.

### 4.4 Chain mediation through induction adaptation and job burnout

While previous studies have identified direct relationships among organizational support, induction adaptation, job burnout, and turnover intentions—as well as the mediating role of job burnout between organizational support and turnover intentions—this study uniquely demonstrates the sequential mediation of induction adaptation and job burnout in the relationship between organizational support and turnover intention. Specifically, organizational support influences induction adaptation among novice teachers in less-developed regions, which in turn affects their level of job burnout, and ultimately predicts their turnover intentions. This finding provides new perspectives for future research and practice.

According to COR theory, individuals strive to retain, protect, and build resources. Potential or actual loss of resources, or even a lack of expected gains, can induce psychological stress. Individuals with abundant resources can invest these resources to acquire new ones, thereby enhancing their capacity to cope with stress and fostering a continuous accumulation of resources—a process known as the “gain spiral.” Conversely, those with scarce resources are not only more vulnerable to the stress caused by resource loss but also often find that their efforts to prevent further depletion lead to even greater deficits, trapping them in a “loss spiral” (Hobfoll, 2001). When resources are exhausted or nearly depleted, individuals may enter a defensive mode, resulting in defensive, aggressive, or even irrational behavioral responses. In this study, organizational support provides novice teachers with valuable resources—such as material conditions and personal traits—that increase the likelihood of entering a “gain spiral” and reduce the risk of falling into a “loss spiral.” This enhances their adaptability, promotes positive emotional experiences, and prevents job burnout caused by resource depletion. As a result, a mutually beneficial exchange of resources between the individual and the organization is fostered, strengthening novice teachers' organizational commitment and embeddedness, and ultimately effectively preventing the defensive reactions that trigger turnover intentions or actual turnover behavior.

## 5 Implications for theory and practice

This study provides new evidence for the literature on the impact of organizational support on teachers' turnover intention, and offers new insights into how organizational support influences novice teachers' turnover intention through induction adaptation and job burnout. Based on these findings, practical recommendations are proposed to mitigate novice teachers' turnover intention in underdeveloped regions.

This study carries important theoretical implications. While existing studies have examined the relationship between organizational support and teachers' turnover intention, the inclusion of induction adaptation and job burnout as mediating variables in this research context has remained underexplored. The findings demonstrate that organizational support significantly and negatively predicts turnover intention among novice teachers in underdeveloped regions, with induction adaptation and job burnout exerting both independent and sequential mediating effects on this relationship. Notably, when job burnout is incorporated as a variable, induction adaptation exerts an overall negative effect on the link between organizational support and turnover intention, alongside a partial positive effect. In other words, induction adaptation may act as a “double-edged sword”: while it can positively influence turnover intention, it exerts a negative effect on turnover intention by inhibiting job burnout. The interplay of positive and negative effects constitutes a suppression effect; given that the negative effect outweighs the positive one, turnover intention is curbed overall. Unlike previous research, which framed the impact of induction adaptation on turnover intention as a simple binary of either positive or negative, this study uncovers the dynamic transition process through which the effect of induction adaptation on turnover intention may shift from negative to positive.

In terms of practical implications, effectively alleviating novice teachers' turnover intention requires efforts to enhance the systematicness and synergy of organizational support. First, schools and educational administrative departments should establish a vertically integrated, systematic support mechanism. Relying solely on schools to slow down the depletion of resources is insufficient; instead, schools and administrative departments must collaborate and align their efforts in terms of the forms, content, and intensity of support. For instance, at the school level, targeted professional development support systems should be designed separately for newly recruited teachers, emerging young teachers, and promising core teachers. At the level of educational administrative departments, a scientific and reasonable progressive incentive mechanism for the evaluation and reward of novice teachers should be established to foster a conducive educational ecosystem that enables teachers to devote themselves to teaching with peace of mind, dedication, and satisfaction. Second, focusing on the key variables in the influence path, we should leverage the synergistic effect of multi-dimensional factors. It is necessary to not only strengthen the role of protective factors such as organizational support and induction adaptation but also control the adverse impact of job burnout. This will prevent induction adaptation from exerting its “detrimental effect”—a risk that arises when negative factors come into play.

## 6 Limitations and future research

Like other studies, this research also has certain limitations. Firstly, although the data on organizational support, induction adaptation, job burnout, and turnover intentions were collected at a relatively concentrated time point, and the findings align with theoretical reasoning and are supported by existing literature, the cross-sectional design cannot provide sufficient evidence for establishing causal relationships. Subsequent studies should adopt longitudinal or experimental designs to address this limitation. Secondly, this study was conducted in three counties in Guizhou Province, China, which may limit the external validity of the findings. Future research could select samples from regions with different cultural backgrounds and employ broader, more diverse samples to enhance the generalizability of the conclusions.

## 7 Conclusions

This study confirms that organizational support significantly and negatively predicts turnover intentions among novice teachers in primary and secondary schools within less-developed regions, indicating that enhancing organizational support can effectively reduce their inclination to leave the profession. Furthermore, the research identifies induction adaptation and job burnout as critical mediating factors, operating through both independent and sequential mediating pathways in the relationship between organizational support and turnover intentions. Of particular note is the finding that induction adaptation may function as a “double-edged sword.” Within the influence of organizational support on turnover intentions, induction adaptation exerts opposing effects across two distinct pathways. In the path “organizational support → induction adaptation → turnover intention,” it exhibits an independent positive effect, suggesting that improved induction adaptation may paradoxically increase the risk of turnover intention. Conversely, in the sequential path “organizational support → induction adaptation → job burnout → turnover intention,” induction adaptation negatively influences turnover intentions by mitigating job burnout. This suppression phenomenon reveals that while these opposing effects counteract each other, the negative pathway predominates, resulting in an overall negative indirect effect. These findings carry significant theoretical and practical implications. Theoretically, this study provides new evidence to reconcile academic debates regarding the dual-effect nature of induction adaptation and offers fresh insights into the “black box” of how its impact on turnover intention may dynamically shift from negative to positive. Practically, education authorities and schools should not only attend to novice teachers' welfare, recognize their contributions, and provide necessary support for high-quality job performance but also prioritize addressing negative factors—such as job burnout—that hinder the positive role of induction adaptation. This approach helps prevent the overall negative effect of organizational support—which reduces turnover intention through enhanced induction adaptation and suppressed job burnout—from being weakened or even reversed.

## Data Availability

The raw data supporting the conclusions of this article will be made available by the authors, without undue reservation.
